# MiR-203a-3p attenuates apoptosis and pyroptosis of chondrocytes by regulating the MYD88/NF-κB pathway to alleviate osteoarthritis progression

**DOI:** 10.18632/aging.205373

**Published:** 2023-12-13

**Authors:** Jiayi Chen, Zhutong Liu, He Sun, Mange Liu, Jiangliang Wang, Chenxiao Zheng, Xuewei Cao

**Affiliations:** 1 Zhongshan Hospital of Traditional Chinese Medicine Affiliated to Guangzhou University of Traditional Chinese Medicine, Zhongshan 528401, Guangdong, China; 2 The Second Clinical College of Guangzhou University of Chinese Medicine, Guangdong Provincial Hospital of Chinese Medicine, Guangzhou 510120, Guangdong China; 3 Liuyang Hospital of Traditional Chinese Medicine, Liuyang 410300, Hunan, China

**Keywords:** miR-203a-3p, osteoarthritis, MYD88, oxidative stress, apoptosis, pyroptosis

## Abstract

Background: Osteoarthritis (OA) is a degenerative joint disease that imposes a significant socioeconomic burden worldwide. Our previous studies revealed a down-regulation of miR-203a-3p in the knee tissues of OA patients. However, the underlying mechanism through which miR-203a-3p mediates the pathological process of OA remains unknown. Thus, we aimed to determine the effects of miR-203a-3p in the progression of OA.

Methods: Rat primary chondrocytes were stimulated with 10 μg/mL lipopolysaccharide (LPS) for 24 hours, followed by transfection with 50 nM miR-203a-3p mimic, inhibitor, and siRNA for MYD88 or consistent negative controls for 48 hours. To evaluate the effects of miR-203a-3p on cartilage matrix degradation, oxidative stress, apoptosis, and pyroptosis in chondrocytes, various techniques such as immunofluorescence staining, biochemical analysis, Western blotting, and the TUNEL staining were utilized. In the rat OA model, all rats were randomly divided into four groups: Sham, OA, OA+Agomir negative control (NC), and OA+Agomir. They received intra-articular injections of 25 nmol miR-203a-3p agomir, agomir NC, or normal saline twice a week for the duration of 8 weeks after OA induction. Immunofluorescence staining was performed to evaluate the effects of miR-203a-3p on cartilage matrix degradation in rats.

Results: MiR-203a-3p was down-regulated in LPS-treated rat chondrocytes and OA cartilage, and directly targeted MYD88. Moreover, miR-203a-3p significantly inhibited LPS-induced cartilage matrix degradation, oxidative stress, apoptosis, and pyroptosis of chondrocytes via targeting MYD88. Mechanistically, miR-203a-3p exerted protective effects via the inhibition of the MYD88/NF-κB pathway. In the rat OA model, intra-articular injections of miR-203a-3p agomir also significantly inhibited cartilage matrix degradation, thereby alleviating OA progression. Furthermore, the miR-203a-3p agomir-treated arthritic rat dramatically exhibited better articular tissue morphology and lower OARSI scores.

Conclusions: MiR-203a-3p plays a role in alleviating the progression of OA by regulating the MYD88/NF-κB pathway, thereby inhibiting cartilage matrix degradation, oxidative stress, apoptosis, and pyroptosis of chondrocytes. It highlights the potential significance of miR-203a-3p as an important regulator of OA.

## INTRODUCTION

Osteoarthritis (OA) is a degenerative joint disease that significantly impacts the quality of life for middle-aged and elderly individuals [1, 2]. It is accompanied by joint pain, swelling, deformity, and mobility impairment, resulting in a substantial socioeconomic burden worldwide [3, 4]. Currently, OA treatment follows a step-therapy approach that involves basic therapy, medication, and surgery [5, 6]. Basic therapies, such as weight loss and exercise, are preferred for mild cases of OA. Medication is widely used to alleviate pain and inflammation in early and middle-stage OA patients [7]. Surgical treatment primarily focuses on pain relief, improving joint function, and correcting deformities [8, 9]. However, the exact mechanisms behind OA pathogenesis remain unclear. Furthermore, current OA treatment methods mainly target symptom relief rather than prevention or progression. Hence, it is crucial to investigate the pathogenesis of OA and explore therapeutic strategies.

Moreover, microRNAs (miRNAs) are small RNAs (17-24 nt) capable of inducing mRNA degradation or suppressing translation [10]. Certain miRNAs play a role in OA biological processes through anti-inflammatory responses, anti-extracellular matrix degradation, anti-oxidative stress, anti-apoptosis, and anti-pyroptosis [11, 12]. Our previous research demonstrated that the expression of miR-203a-3p in OA knee tissue was decreased by high-throughput sequencing of miRNA [13]. However, the precise mechanism through which miR-203a-3p mediates OA pathogenesis remains unclear. Therefore, elucidating the regulatory mechanisms of miR-203a-3p could contribute to discovering therapeutic targets.

The nuclear factor-kappa B (NF-κB) pathway has been studied to promote the inflammatory response in OA development [14]. Activation of this pathway leads to the production of pro-inflammatory cytokines such as interleukin-1β (IL-1β) and TNFα, triggering inflammatory response and exacerbating OA progression [14, 15]. MYD88 acts as a typical adaptor for downstream inflammatory signaling pathways of Toll-like receptor (TLR) and IL-1 receptor family members. It regulates the NF-κB pathway through NF-κB factor phosphorylation [16, 17]. Nevertheless, the potential regulatory mechanisms of MYD88 and the NF-κB pathway in OA remain unknown.

Hence, this study was aimed to explore the effects of the miR-203a-3p/MYD88/NF-κB pathway on the progression of OA. Our findings might shed light on the role of miRNAs and the MYD88/NF-κB pathway in the context of OA and have significant implications for the development of new therapeutic strategies.

## MATERIALS AND METHODS

### Primary culture of rat chondrocytes

Primary chondrocytes were obtained from 2-week-old neonate rats and identified as previously described [18]. Under suitable conditions, primary chondrocytes were cultured in Dulbecco’s Modified Eagle Medium (DMEM; Gibco) containing 10% fetal bovine serum (FBS; Gibco). The second generation of chondrocytes was planted on the plate for the next experiment.

### Quantitative real-time polymerase chain reaction (qRT-PCR)

Total RNA was extracted from rat primary chondrocytes using an RNAiso Plus Kit. Reverse transcription was performed with the PrimeScript RT kit. Both kits were purchased from Takara Biotechnology, Japan. The qPCR analyses were performed with the SYBR^®^ qPCR Kit (Accurate Biotechnology) The downstream primers of miR-203a-3p were constructed using a universal downstream primer kit. The reaction conditions were set using a two-step PCR reaction program. The mRNA expression of miR-203a-3p and MYD88 were assessed using the 2^−ΔΔCt^ method. The primer sequences were shown in Table 1. The methods of Zhen Li et al. 2022 were followed [19].

**Table 1:** Primer sequences for qRT-PCR.

**Gene**	**Primer sequence**
miR-203a-3p	5’-TGTGCGGAGTGAAATGTTTAGGA-3’
U6	F:5’-GCTTCGGCAGCACATATACTAAAAT-3’
	R:5’-CGCTTCACGAATTTGCGTGTCAT-3’
MYD88	F:5’-GTCTCCAGGTGTCCAACAGAAGC-3’
	R:5’-GTCGCAGATAGTGATGAACCGTAGG-3’
GAPDH	F:5’-ACCCAGAAGACTGTGGATGG-3’
	R:5’-GAGGCAGGGATGATGTTCTG-3’

Abbreviations: F, forward; R, reverse.

### Western blot (WB)

Rat primary chondrocytes were sonicated in radioimmunoprecipitation assay (RIPA) lysis buffer (Gibco, Grand Island, NY, USA). The protein concentration of different groups should be normalized. The protein was separated by electrophoresis and blocked by 5% skim milk after transmembrane. After that, the membranes were incubated overnight with primary antibodies targeting MYD88 (Abcam), matrix metallopeptidase 3 (MMP3, Proteintech), MMP13 (Proteintech), ADAMTS-5 (Abcam), collagen II (Abcam), SOX9 (Proteintech), Aggrecan (Abcam), Bcl-2 (CST), Bax (CST), cleaved caspase-3 (CST), NLR Family Pyrin Domain Containing 3 (NLRP3, Affinity), apoptosis-associated speck-like protein containing a CARD (ASC, CST), cleaved caspase-1 (CST), gasdermin D (GSDMD, Affinity), IL-1β (CST), IL-18 (CST), phosphorylated IκBα (p-IκBα, Affinity), IκBα (Affinity), p-p65 (CST), p65 (CST), and GAPDH (CST) at 4° C. Dilute the primary antibody at 1:1000. After incubation by secondary antibody (1:1000) for 90 minutes, ECL luminescent solution exposed WB bands. We followed our previous methods [19].

### Bioinformatics analysis and luciferase reporter assay

The miWALK, Tarbase v.8, and miTarbase online databases were performed to predict miR-203a-3p target genes. DNA fragments containing normal and mutant binding sites of miR-203a-3p in the 3’-untranslated region (3’-UTR) of MYD88 were inserted into the luciferase reporter gene plasmid to obtain MYD88-WT (wild type) and MYD88-MUT (mutant) plasmids, respectively. Subsequently, miR-203a-3p mimics and plasmids were co-transfected into 293T cells. Finally, luciferase activity was determined.

### Transfection and treatment of chondrocytes

Two days before transfection, rat primary chondrocytes were seeded into a six-well plate (2x10^5^ cells/well). After that, chondrocytes were transfected with 50 nM miR-203a-3p mimic, inhibitor, and siRNA for MYD88, or consistent negative controls (RIBO Biotechnology Company) using the Lipofectamine 2000 reagent. After 48 hours, the effects of cell transfection were analyzed by qRT-PCR. Subsequently, the cells were stimulated with 10 μg/mL lipopolysaccharide (LPS) (Sigma-Aldrich, St Louis, MO, USA) for 24 hours and then harvested.

### TUNEL assay

TUNEL staining was performed by the Apoptosis Detection Kit (Yeasen Biotech, Shanghai, China) [20, 21]. The cells were fixed for 25 minutes and permeabilized with proteinase K (20 μg/ml) for 20 minutes. After incubating with TdT for 1 hour, the cells were counterstained with 4’,6-diamidino-2-phenylindole (DAPI). Finally, the number and proportion of apoptotic cells were calculated. Our previous methods were followed [19].

### Oxidative stress analysis

Malondialdehyde (MDA) and superoxide dismutase (SOD) activity in chondrocytes were detected using kits (Jiancheng Institute of Biology, China) after LPS treatment and transfection. The liquid supernatant was collected for measurement according to our previous methods [19]. The cellular reactive oxygen species (ROS) levels were measured using the 2,7-dichlorodihydrofluorescein diacetate (2,7-DCF-DA) staining kit (Sigma-Aldrich). The cells were washed twice with phosphate buffered saline (PBS) and incubated with 10 μM 2’,7’-dichlorofluorescin diacetate (DCFH-DA) fluorescent probe at 37° C for 30 minutes. Finally, the stained cells were calculated. The methods of Zhen Li et al. 2022 were followed [19].

### Establishment of the OA model and treatment

The OA model was created by surgically inducing anterior cruciate ligament transection (ACLT) as previously described [22, 23]. In the sham group, rats received only the incision without surgical ACLT. All animals were divided into four groups: sham-operated, OA, OA+Agomir negative control (NC), and OA+Agomir groups (n = 12 per group). In the OA+Agomir and OA+Agomir NC groups, rats received intra-articular injections of 25 nmol miR-203a-3p agomir or agomir NC, respectively, twice a week for 8 weeks after OA induction. A volume of 20 μL of the solution was injected into the right knee. In the sham-operated and OA groups, rats received an equal volume of normal saline.

### Histological assessment

After eight weeks of treatment with miR-203a-3p agomir, the animals were euthanized. Right knee tissue was fixed and decalcified, then paraffin embedded and sected. Subsequently, the sections were dewaxed and stained with different kits, and sealed. We followed the methods reported by Zhen Li et al. in 2022 [19]. The Osteoarthritis Research Society International (OARSI) scoring system were performed [24]. Hematoxylin-eosin (HE), Toluidine blue, and Safranin O-Fast Green staining were performed following standard protocols [25]. The sections were observed using an Olympus IX73 microscope.

### Immunofluorescence staining

Immunofluorescence staining was conducted following standard protocols and our previous methods [19–21]. Chondrocytes that had undergone suitable treatment were fixed with 4% PFA for 30 minutes and permeabilized in 0.3% Triton X-100 at room temperature (RT) for 30 minutes. After incubating with 10% FBS for 1 hour at RT to block nonspecific binding, the samples were incubated overnight with primary antibodies against MYD88 (1:200, Abcam), collagen II (1:200, Abcam), MMP13 (1:200, Proteintech), NLRP3 (1:200, Affinity), GSDMD (1:100, Affinity), p-IκBα (1:200, Affinity), and p-p65 (1:200, CST) at 4° C. Next, the samples were incubated with secondary antibodies (1:300, Invitrogen). Finally, the fluorescence intensity of positive cells was observed under fluorescence microscope.

### Statistical analysis

Statistical analyses were performed using SPSS 20.0 software (SPSS, Chicago, IL, USA). Data are presented as mean ± standard deviation (SD). One-way analysis of variance (ANOVA) was used to analyze multiple groups, and unpaired Student’s *t*-test was used to analyze two groups. *P* < 0.05 was considered statistically significant.

### Data availability

The data used to support the findings of this study are available from the corresponding author upon reasonable request.

## RESULTS

### miR-203a-3p was decreased, and MYD88 was increased in LPS-treated chondrocytes

**Figure 1:**
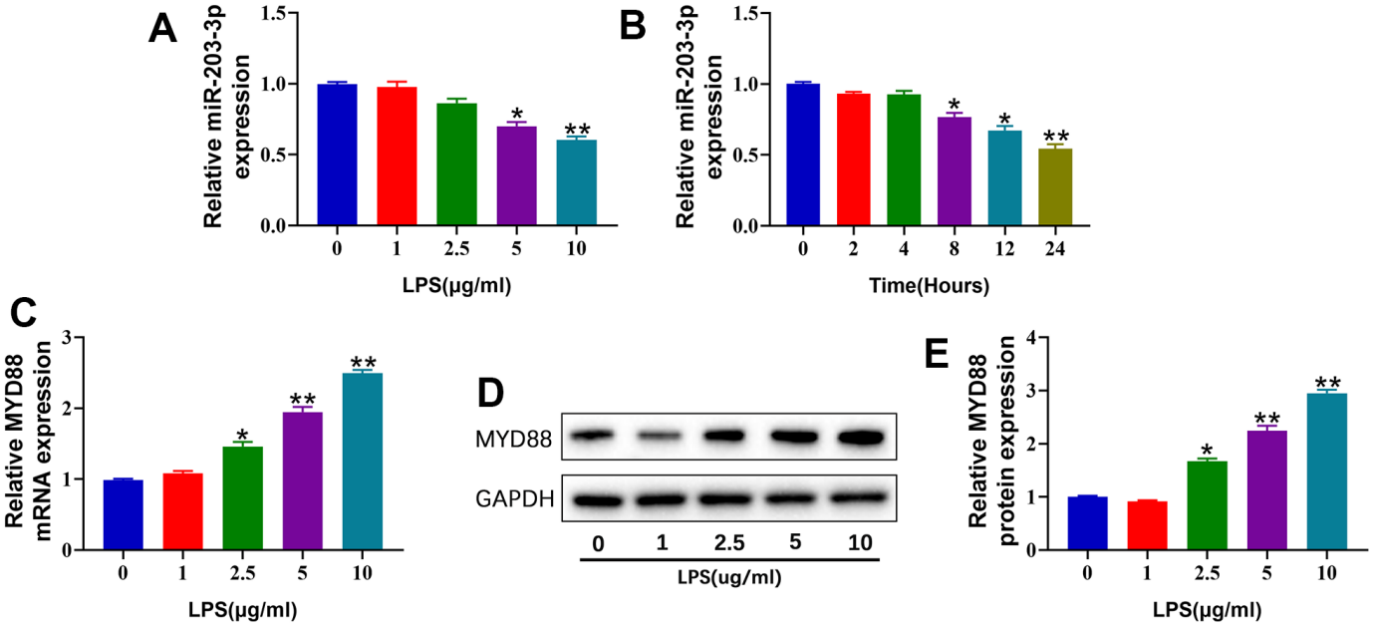
**miR-203a-3p was decreased, and MYD88 was increased in LPS-treated chondrocytes.** (**A**, **B**) qPCR showing miR-203a-3p expression in primary rat chondrocytes treated with different concentration of LPS (0, 1, 2.5,5 and 10 μg/ml) for 24 hours and treated with 10 μg/ml at different time points (0, 2,4,8,12 and 24 hours). (**C**) qPCR showing MYD88 mRNA expression in primary rat chondrocytes treated with different concentration of LPS for 24 hours. (**D**, **E**) WB analysis and relative quantification showing the protein expression of MYD88 in primary rat chondrocytes treated with different concentration of LPS for 24 hours. All experiments were performed in triplicated and data were presented as the mean±SD, *n*=3 per group. *P<0.05, **P<0.01.

To investigate the effects of LPS on the expression of miR-203a-3p in chondrocytes, we stimulated primary chondrocytes with increasing concentrations of LPS (0-10 μg/mL) for different durations (0-24 hours). qPCR analysis revealed that LPS stimulation significantly reduced miR-203a-3p expression in chondrocytes in a dose- and time-dependent manner (Figure 1A, 1B). Consequently, we used a treatment of 10 μg/mL LPS for 24 hours to establish the OA model of chondrocytes in subsequent experiments. In contrast, the expression of MYD88 was substantially upregulated in chondrocytes after LPS stimulation, as verified by qPCR and WB (Figure 1C–1E). These findings indicated that LPS stimulation downregulated miR-203a-3p and upregulated MYD88 expression in chondrocytes.

### miR-203a-3p directly targeted MYD88

**Figure 2:**
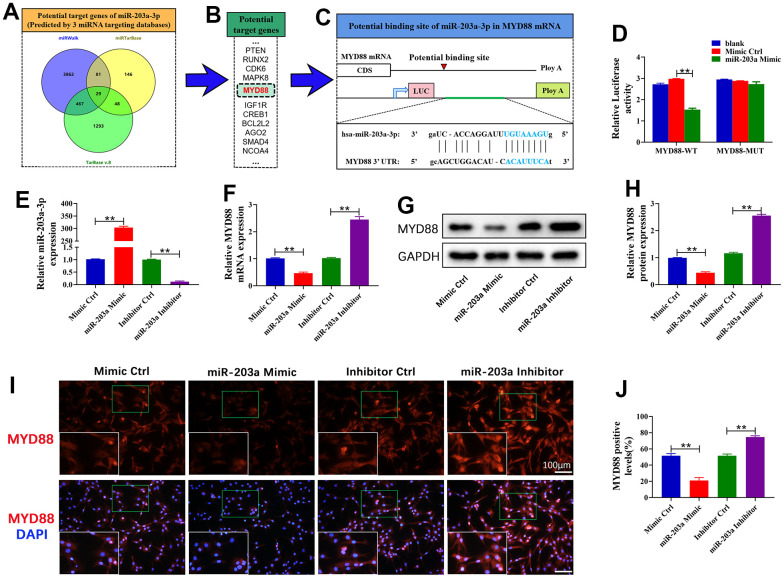
**miR-203a-3p directly targeted MYD88.** (**A**, **B**) Three online databases were used to predict the miR-203a-3p target genes. (**C**) Schematic representation of the MYD88 3’-UTR. A putative miR-203a-3p-binding site existed in the MYD88 mRNA. (**D**) The relative luciferase activity between cells was compared following co-transfection of MYD88-WT/ MYD88-MUT vectors with miR-203a-3p mimics and consistent negative controls. (**E**, **F**) qRT-PCR showing the mRNA expression of miR-203a-3p and MYD88 following transfection with miR-203a-3p mimic, inhibitor and consistent negative controls. (**G**–**J**) WB and immunofluorescence analysis showing the protein expression of MYD88 following transfection. All experiments were performed in triplicated and data were presented as the mean±SD, *n*=3 per group. *P<0.05, **P<0.01.

The online databases identified MYD88 as a target gene of miR-203a-3p (Figure 2A–2C). Thus, we constructed WT and MUT MYD88 3’-UTR fragments to investigate whether miR-203a-3p targets MYD88. The results showed that the miR-203a-3p mimic did not affect the luciferase activity of MYD88-MUT 293T cells but dramatically inhibited the activity of MYD88-WT 293T cells (Figure 2D). These data demonstrated that miR-203a-3p directly targets MYD88. Furthermore, to assess the regulatory relationship between miR-203a-3p and MYD88, chondrocytes were transfected with the miR-203a-3p mimic, inhibitor, and consistent negative controls. The results showed that the expression of miR-203a-3p was dramatically increased after transfection with the miR-203a-3p mimic, indicating successful transfection of chondrocytes (Figure 2E). Moreover, MYD88 mRNA dramatically decreased in miR-203a-3p mimic-treated chondrocytes. In contrast, the miR-203a-3p inhibitor dramatically upregulated MYD88 expression (Figure 2F). Consistent trends were observed in MYD88 protein levels as detected by WB and immunofluorescence (Figure 2G–2J). The above results revealed that miR-203a-3p downregulated MYD88.

### miR-203a-3p inhibited LPS-induced cartilage matrix degradation and apoptosis in chondrocytes

**Figure 3:**
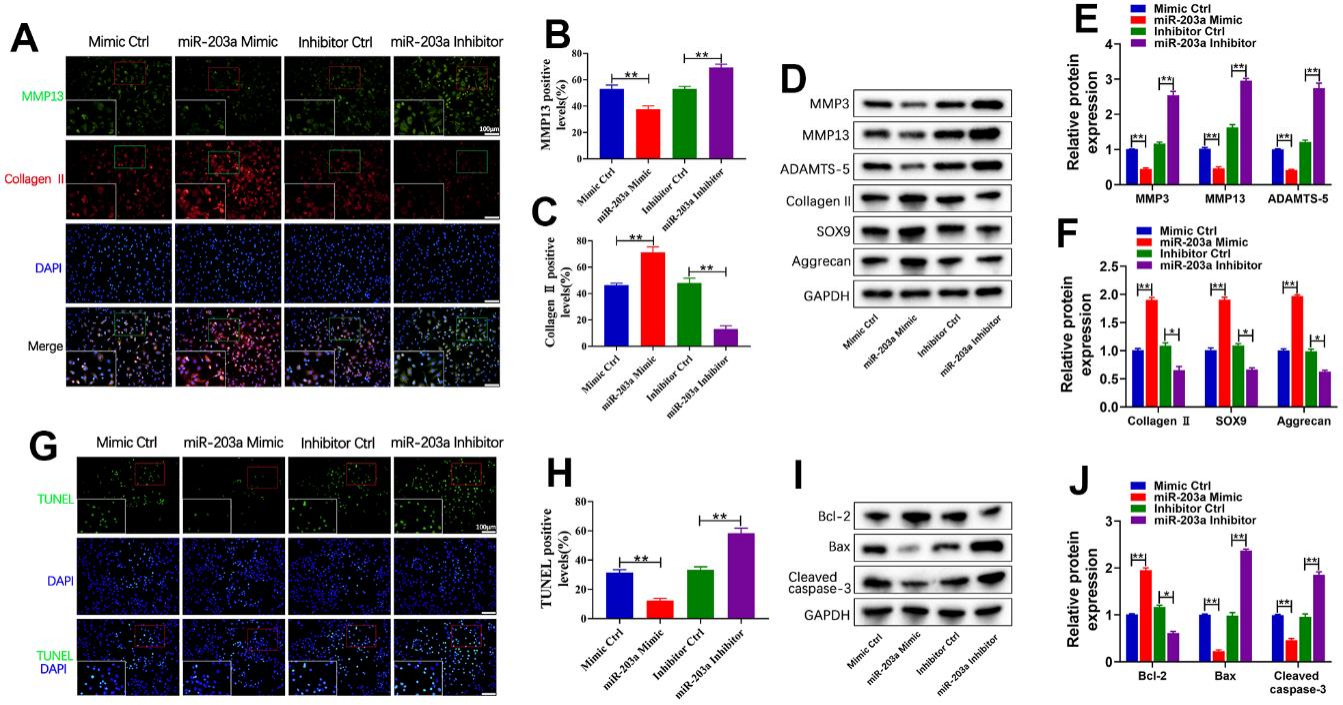
**miR-203a-3p inhibited LPS-induced cartilage matrix degradation and apoptosis in chondrocytes.** (**A**–**C**) Co-immunofluorescence staining of MMP13 (green) / Collagen II (red) and the relative quantification of positive cells in LPS-induced chondrocytes after transfection with miR-203a-3p mimic, inhibitor and consistent negative controls. (**D**–**F**) WB analysis and the relative quantification showing the expression levels of cartilage matrix degradation related proteins (MMP3, MMP13, ADAMST-5, Collagen II, SOX9 and Aggrecan). (**G**, **H**) TUNEL staining showing the chondrocyte apoptosis and quantitative estimation of the number of TUNEL positive cells. (**I**, **J**) WB analysis and the relative quantification showing the expression levels of apoptosis related proteins (Bcl-2, Bax, and Cleaved caspase-3). All experiments were performed in triplicated and data were presented as the mean±SD, *n*=3 per group. *P<0.05, **P<0.01.

To assess the effects of miR-203a-3p against cartilage matrix degradation and cell apoptosis, primary chondrocytes were stimulated with 10 μg/mL of LPS for 24 hours. Subsequently, these cells were treated with overexpression or inhibited expression of miR-203a-3p. Double immunofluorescence analysis revealed a significant decrease in MMP13 expression and an increase in collagen II expression within the overexpression group. Meanwhile, compared to controls, treatment with the miR-203a-3p inhibitor demonstrated an upregulation of MMP13 and a downregulation of collagen II expression (Figure 3A–3C). Additionally, WB results further confirmed that treatment with miR-203a-3p mimic decreased MMP3, MMP13, and ADAMTS-5 levels but increased collagen II, SOX9, and aggrecan levels (Figure 3D–3F). Collectively, these data demonstrated that overexpression of miR-203a-3p effectively counteracts cartilage matrix degradation.

Moreover, the TUNEL staining demonstrated a notable decrease in the number of apoptotic cells following miR-203a-3p overexpression. Conversely, inhibition of miR-203a-3p resulted in contrasting outcomes (Figure 3G, 3H). Similarly, miR-203a-3p overexpression resulted in a significant upregulation of the anti-apoptotic Bcl-2 protein, while the pro-apoptotic Bax and cleaved caspase-3 proteins were downregulated when compared to controls (Figure 3I, 3J). The above data showed that miR-203a-3p effectively inhibited LPS-induced degradation of cartilage matrix and apoptosis of chondrocytes *in vitro*.

### miR-203a-3p inhibited LPS-induced pyroptosis and oxidative stress in chondrocytes

**Figure 4:**
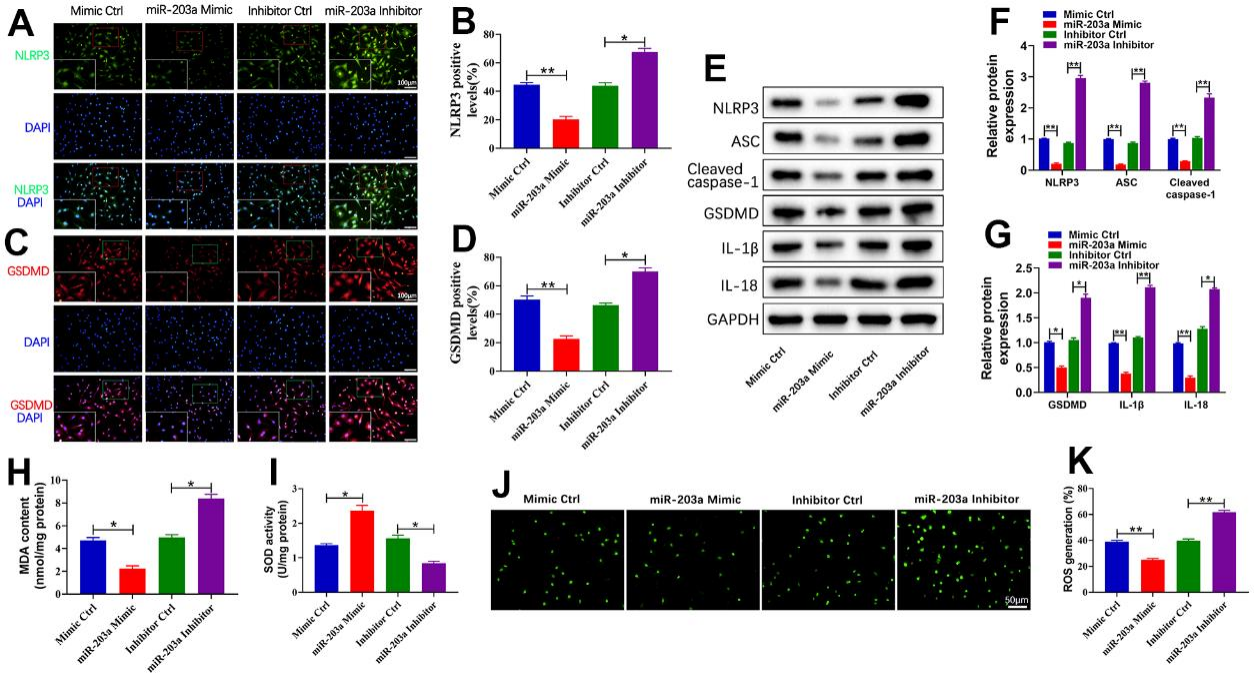
**miR-203a-3p inhibited LPS-induced pyroptosis and oxidative stress in chondrocytes.** (**A**–**D**) Immunofluorescence staining of NLRP3 (green) and GSDMD (red) as well as the relative quantification of positive cells were performed respectively in LPS-induced chondrocytes following transfection with miR-203a-3p mimic, inhibitor and consistent negative controls. (**E**–**G**) WB analysis and the relative quantification showing the expression levels of pyroptosis related proteins (NLRP3, ASC, Cleaved caspase-1, GSDMD, I L-1β, and IL-18). (**H**, **I**) Malondialdehyde (MDA) levels and superoxide dismutase (SOD) activity in LPS-induced chondrocytes following transfection. (**J**, **K**) ROS production in LPS-induced chondrocytes after transfection was measured by DCF staining. All experiments were performed in triplicated and data were presented as the mean±SD, *n*=3 per group. *P<0.05, **P<0.01.

Pyroptosis is a process mediated by NLRP3 and caspase-1 that releases inflammatory factors, promoting the inflammatory response and ultimately resulting in cell death [26, 27]. To assess the effects of miR-203a-3p overexpression or inhibition on LPS-induced pyroptosis in chondrocytes, we analyzed the expression of pyroptosis-related proteins. Immunofluorescence analysis revealed a significant decrease in the number of positive NLRP3 and GSDMD cells upon miR-203a-3p overexpression, whereas the treatment with miR-203a-3p inhibition led to their downregulation compared to controls (Figure 4A–4D). Furthermore, WB analysis confirmed that the miR-203a-3p mimic dramatically downregulated pyroptosis-related proteins, whereas the miR-203a-3p inhibitor upregulated these proteins in comparison with controls (Figure 4E–4G).

Moreover, miR-203a-3p overexpression leads to a significant decrease in MDA expression and an increase in SOD expression. Conversely, miR-203a-3p inhibition treatment reversed these trends compared to controls (Figure 4H, 4I). Additionally, analysis of dichlorofluorescein (DCF) suggested that miR-203a-3p overexpression dramatically reduced the levels of ROS production (Figure 4J, 4K). Therefore, miR-203a-3p exhibited the ability to mitigate OA progression by inhibiting LPS-induced pyroptosis and oxidative stress in chondrocytes.

### miR-203a-3p inhibited the NF-κB signaling pathway in chondrocytes

**Figure 5:**
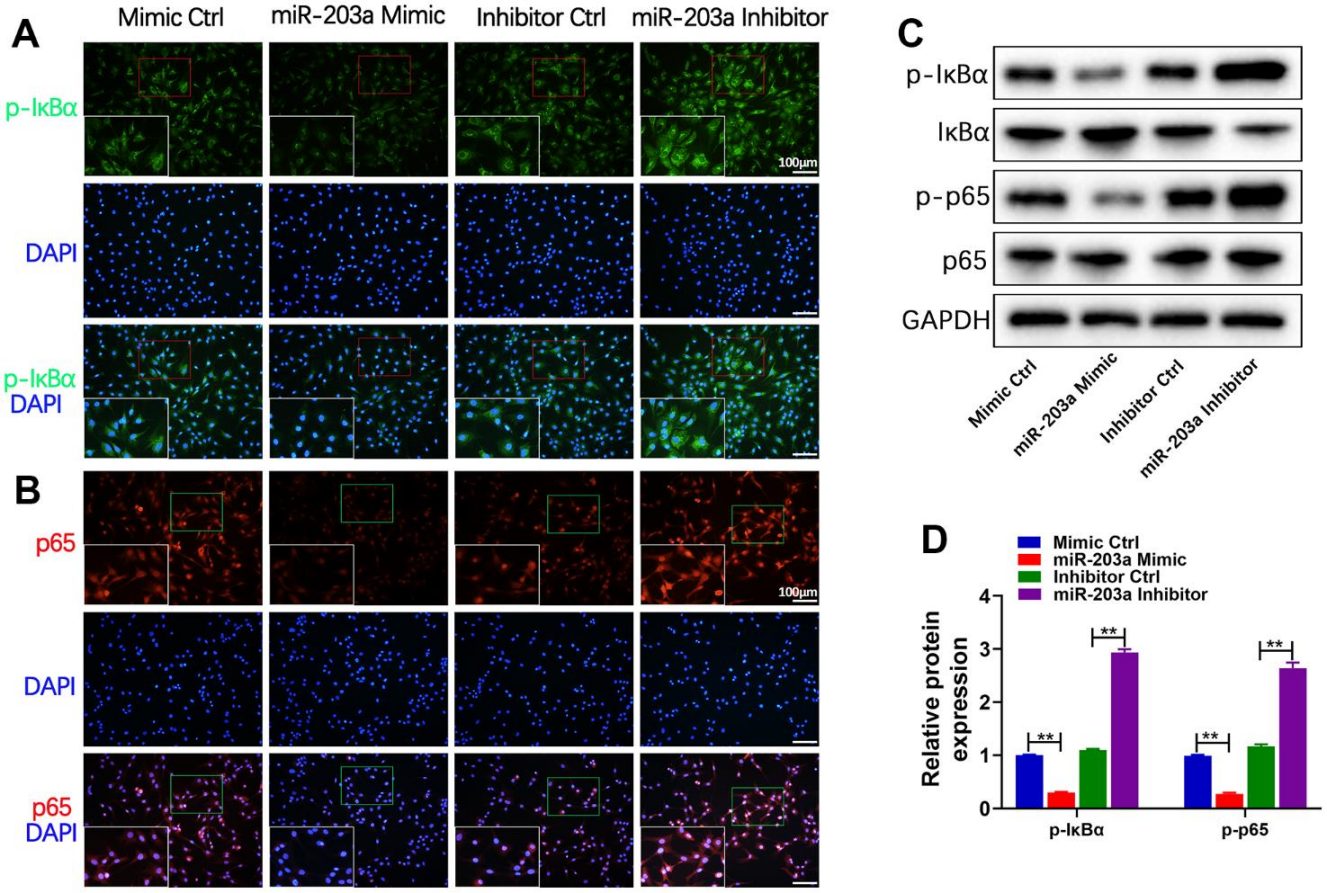
**miR-203a-3p inhibited the NF-κB signaling pathway in chondrocytes.** (**A**, **B**) Immunofluorescence staining showing the phosphorylation of IκBα (green) and the nuclear translocation of p65 (red) in LPS-induced chondrocytes following transfection with miR-203a-3p mimic, inhibitor and consistent negative controls. (**C**, **D**) WB analysis and the relative quantification showing the expression levels of proteins related with NF-κB signaling pathway (p-IκBα, IκBα, p-p65 and p65). All experiments were performed in triplicated and data were presented as the mean±SD, *n*=3 per group. *P<0.05, **P<0.01.

The activation of the IKK complex leads to subsequent translocation of NF-κB dimers to the nucleus for transcriptional regulation of target genes [28, 29]. To elucidate the effects of miR-203a-3p on the NF-κB pathway, the expression of p-IκBα and the nuclear translocation of p65 were detected in chondrocytes after transfection with the miR-203a-3p mimic and inhibitor. Immunofluorescence analysis demonstrated that miR-203a-3p overexpression significantly decreased p-IκBα expression (Figure 5A) and inhibited the nuclear translocation of p65 (Figure 5B) in comparison with controls. Furthermore, WB analysis confirmed that miR-203a-3p overexpression decreased IκBα and p65 phosphorylation, thereby inactivating the NF-κB pathway without affecting the total p65 levels (Figure 5C, 5D). On the contrary, miR-203a-3p inhibition promoted the activation of the NF-κB pathway. Altogether, the above results indicated that miR-203a-3p could effectively suppress the NF-κB pathway in LPS-stimulated chondrocytes.

### miR-203a-3p exerted biological functions on chondrocytes by targeting MYD88

**Figure 6:**
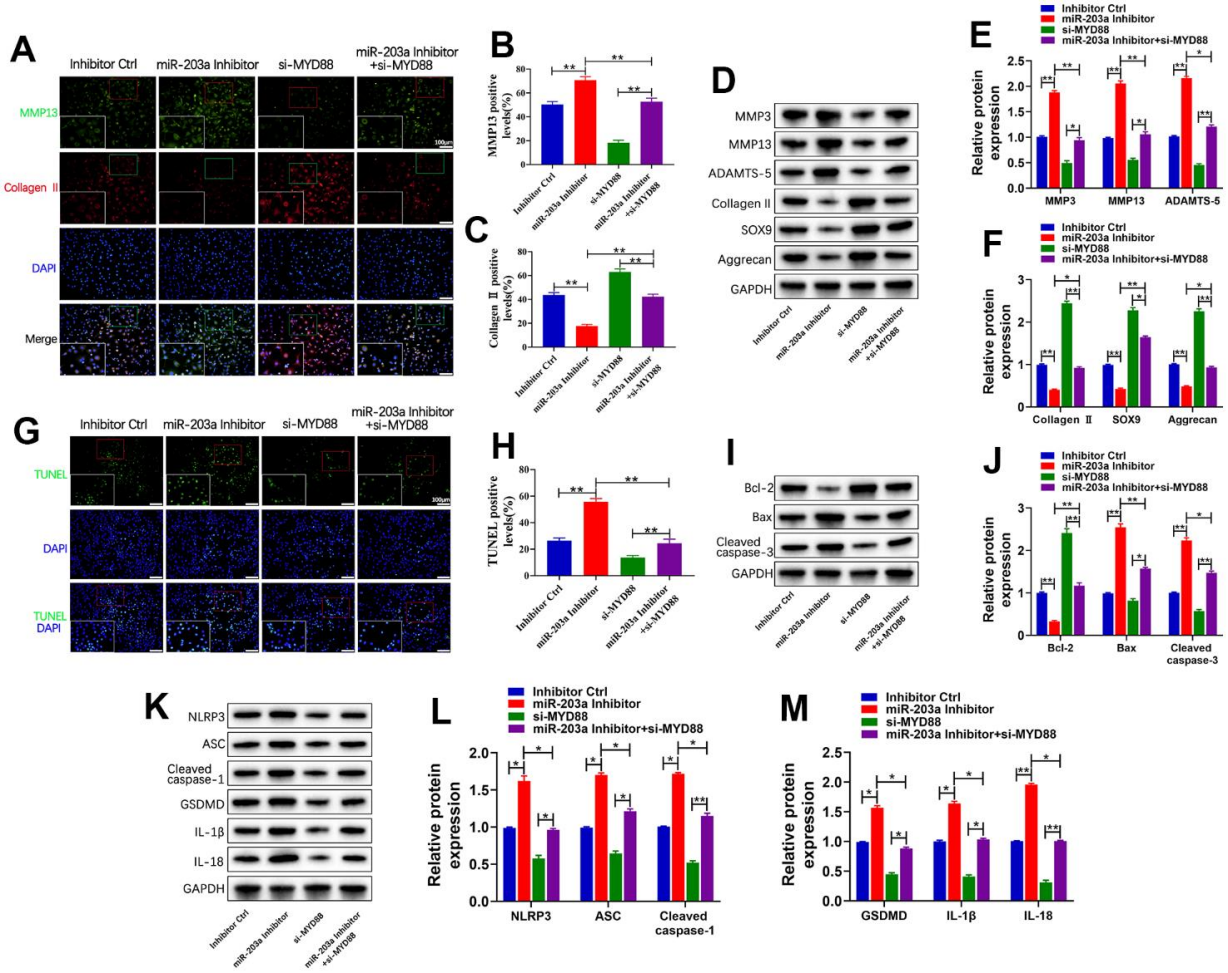
**miR-203a-3p exerted biological functions on chondrocytes by targeting MYD88.** (**A**–**C**) Co-immunofluorescence staining of MMP13 (green) / Collagen II (red) and the relative quantification of positive cells in LPS-induced chondrocytes following transfection with miR-203a-3p inhibitor, si-MYD88, or co-transfection. (**D**–**F**) WB analysis and relative quantification showing the expression levels of cartilage matrix degradation related proteins (MMP3, MMP13, ADAMST-5, Collagen II, SOX9 and Aggrecan). (**G**, **H**) TUNEL staining showing the chondrocyte apoptosis and quantitative estimation of the number of TUNEL positive cells. (**I**, **J**) WB analysis and relative quantification showing the expression levels of apoptosis-related proteins (Bcl-2, Bax and Cleaved caspase-3) after transfection. (**K**–**M**) WB analysis and the relative quantification showing the expression levels of pyroptosis related proteins (NLRP3, ASC, Cleaved caspase-1, GSDMD, IL-1β, and IL-18). All experiments were performed in triplicated and data were presented as the mean±SD, *n*=3 per group. *P<0.05, **P<0.01.

Subsequently, rescue experiments were performed to elaborate the underlying mechanisms through which miR-203a-3p exerts its biological functions. Chondrocytes were transfected with the miR-203a-3p inhibitor or si-MYD88 to inhibit miR-203a-3p and MYD88 expression, respectively. Double immunofluorescence analysis indicated that miR-203a-3p inhibition markedly increased MMP13 levels and decreased collagen II compared to controls. In contrast, MYD88 inhibition decreased MMP13 levels and increased collagen II. As expected, co-transfection with the miR-203a-3p inhibitor and si-MYD88 reversed the inhibitory effect of miR-203a-3p (Figure 6A–6C). Therefore, MYD88 inhibition can rescue the suppressive effect of miR-203a-3p on LPS- induced cartilage matrix degradation. This effect was further confirmed by WB analysis of proteins associated with cartilage matrix anabolism and catabolism (Figure 6D–6F).

Additionally, the number of apoptotic cells markedly increased following miR-203a-3p inhibition treatment compared to controls. However, co-transfection with the miR-203a-3p inhibitor and si-MYD88 markedly reversed this trend (Figure 6G, 6H). Consistently, WB analysis confirmed that MYD88 inhibition could rescue the suppressive effect of miR-203a-3p, resulting in increased Bcl2 expression and decreased Bax and cleaved caspase-3 levels (Figure 6I, 6J). Moreover, co-transfection with the miR-203a-3p inhibitor and si-MYD88 reversed the effects of miR-203a-3p inhibition on the upregulation of pyroptosis-related proteins (Figure 6K–6M). Overall, the above findings showed that miR-203a-3p negatively modulated MYD88 to inhibit LPS-induced cartilage matrix degradation, apoptosis, and pyroptosis in chondrocytes.

### miR-203a-3p alleviated OA progression in ACLT rat models

**Figure 7:**
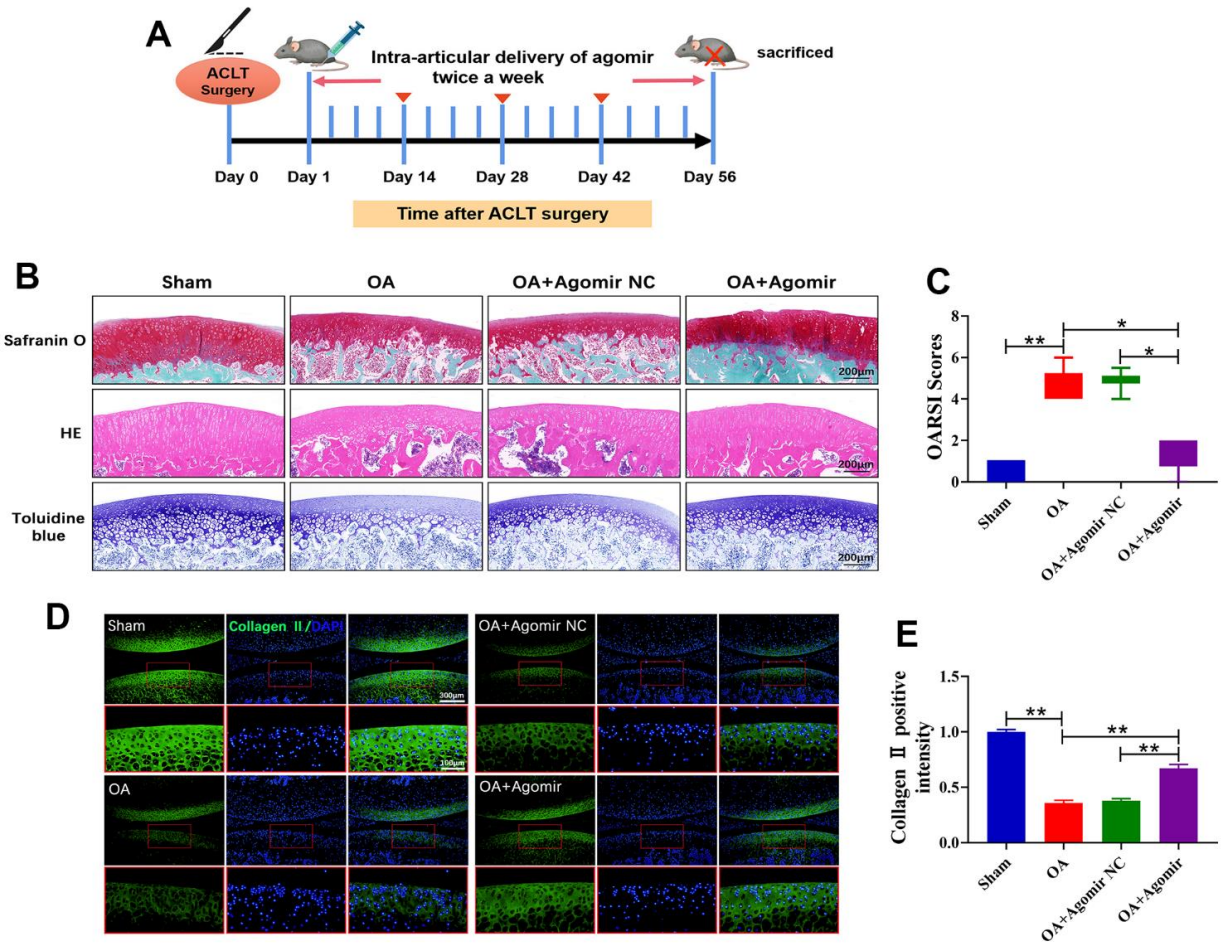
**miR-203a-3p alleviated the destruction of articular cartilage in ACLT rats.** (**A**) ACLT rat model and miR-203a-3p agomir treatment protocol. (**B**) Safranin O-Fast Green, HE, and Toluidine blue staining of rat knee joints were performed to assess the histological morphology of the different groups at 8 weeks after miR-203a-3p agomir treatment. (**C**) The OARSI scores of the different groups were calculated respectively at 8 following after miR-203a-3p agomir treatment. (**D**, **E**) Immunofluorescence staining of Collagen II (green) and MMP13 (red) were performed in rat articular cartilage at 8 weeks following miR-203a-3p agomir treatment. All experiments were performed in triplicated and data were presented as the mean±SD, *n*=3 per group. *P<0.05, **P<0.01.

To demonstrate the effects of miR-203a-3p on OA progression *in vivo*, rats were subjected to intra-articular injections of 25 nmol miR-203a-3p agomir or agomir NC (Figure 7A). Histological staining of knee joint sections from OA rats using Safranin O-Fast Green, HE and Toluidine blue indicated that ACLT surgery induced articular cartilage destruction and erosion. However, miR-203a-3p overexpression attenuated this erosive effect (Figure 7B). Moreover, the OARSI scores were dramatically decreased in the miR-203a-3p agomir-treated rats in comparison with controls, confirming that miR-203a-3p overexpression reversed OA-associated cartilage damage (Figure 7C).

**Figure 8:**
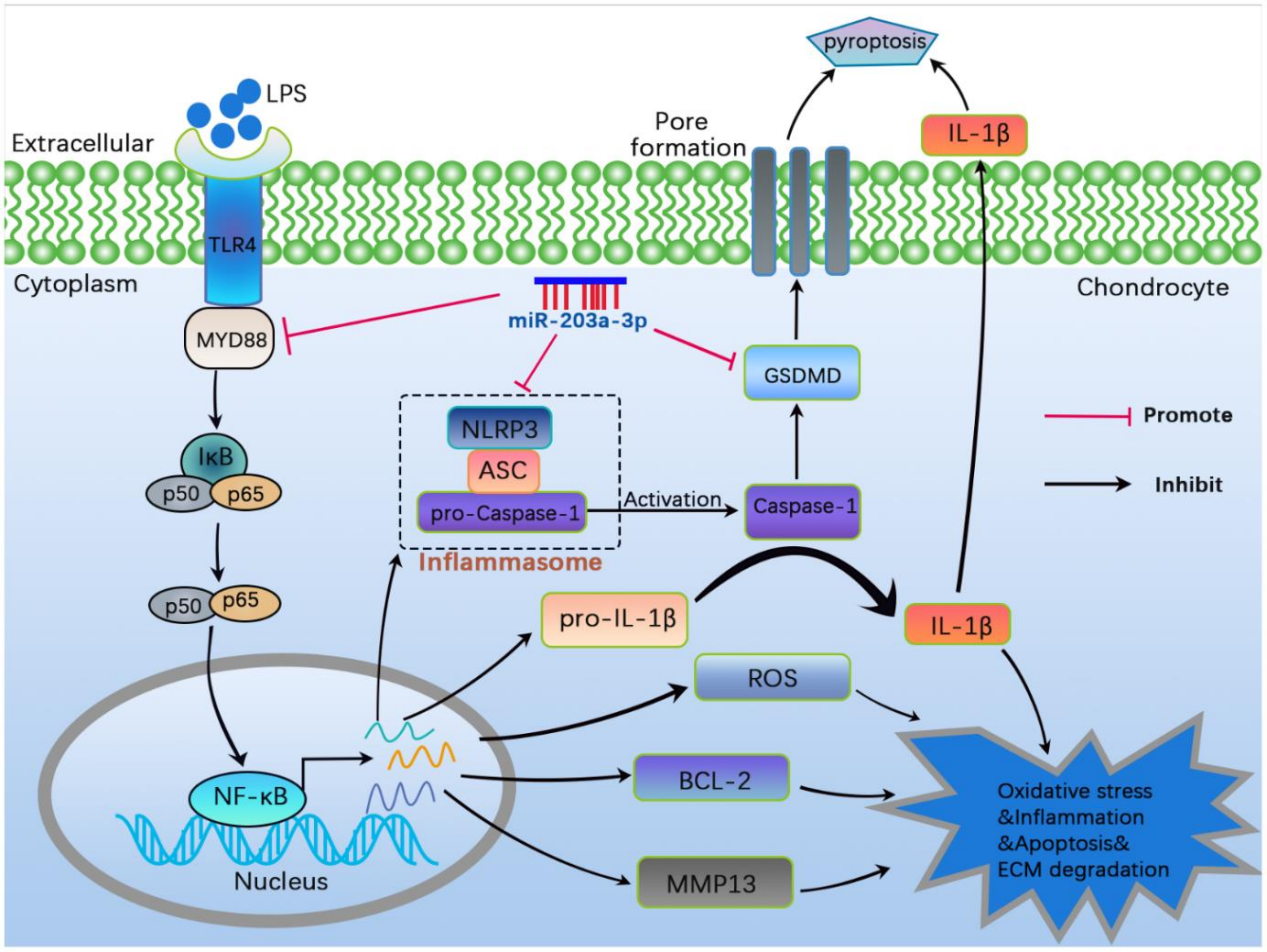
**A schematic diagram illustrating the theory in the current study.** MiR-203a-3p inhibited cartilage matrix degradation as well as alleviated apoptosis and pyroptosis in *in vitro* and *in vivo* OA models by regulating the MYD88/NF-κB pathway.

Consistent with the gross observations, immunofluorescence analysis revealed a significant decrease in collagen II deposition in OA rats. However, treatment with miR-203a-3p overexpression markedly upregulated collagen II levels (Figure 7D, 7E). Thus, miR-203a-3p may alleviate OA progression by regulating cartilage matrix metabolism *in vivo*. Finally, a schematic diagram illustrating the theory presented in Figure 8.

## DISCUSSION

OA is a pathological condition characterized by an imbalance between anabolism and catabolism, resulting in the destruction of articular cartilage [30]. Current treatments for OA provide only temporary relief and are not effective in preventing or slowing the progression of the disease [5, 6, 31], thus it is essential to explore the pathogenesis of OA and develop new therapeutic strategies to ameliorate disease progression.

Previous studies have indicated that miR-203a-3p exhibited anti-inflammatory, anti-oxidant, anti-apoptotic, and anti-pyroptotic effects in various disease models. For instance, in diabetic nephropathy, miR-203a-3p regulates SOX6 to inhibit the inflammatory response of mesangial cells [32]. Additionally, miR-203a-3p has neuroprotective effects by inhibiting apoptosis, NLRP3 inflammasome activity, oxidative stress, and inflammation in hippocampal neurons in ischemic stroke models [33]. In a study on inflammatory bowel disease, miR-203a-3p mediates caspase 11/4-induced macrophage pyroptosis of THP-1 cells in a colitis model [34]. Moreover, miR-203a-3p overexpression promotes apoptosis in ovarian and pancreatic cancer cells through different biological pathways [35, 36]. However, the effects of miR-203a-3p on chondrocytes in the context of OA remain unknown. This study highlighted the biological significance of miR-203a-3p in protecting cartilage against degradation to alleviate OA progression. We also observed that miR-203a-3p suppressed cartilage matrix degradation, apoptosis, and pyroptosis in chondrocytes using both *in vitro* and *in vivo* OA models by targeting MYD88. Thus, our study was the first to shed light on the potential therapeutic role of miR-203a-3p in OA.

The key to the pathological process of OA lies in matrix-degrading enzymes that mediate cartilage destruction. Maintaining the balance between physiological catabolism and anabolism in cartilage is crucial for preventing and alleviating OA [37]. In our study, the results suggested that miR-203a-3p overexpression treatment upregulated the expression of matrix proteins collagen II, SOX9, and aggrecan while downregulating MMP3, MMP13, and ADAMTS-5 in LPS-stimulated chondrocytes. Therefore, miR-203a-3p mitigates OA progression by inhibiting cartilage matrix degradation. The above results were consistent with previous studies indicating that specific miRNAs play a significant role in regulating cartilage matrix metabolism during the pathological process of OA [37–39].

Apoptosis of chondrocytes is often considered a trigger for cartilage degeneration. Furthermore, chondrocyte apoptosis and cartilage matrix damage can form a vicious cycle, exacerbating each other and disrupting cartilage homeostasis [40, 41]. MiR-203a-3p has previously been reported to mediate cell apoptosis in various disease models [36, 42, 43]. Our study yielded similar results, demonstrating that miR-203a-3p overexpression exerts anti-apoptotic effects by increasing Bcl-2 expression and decreasing Bax and cleaved caspase-3 levels. Hence, miR-203a-3p alleviates OA progression by inhibiting chondrocyte apoptosis, which aligns with the neuroprotective effects of miR-203a-3p observed in inhibiting hippocampal neuron apoptosis [33].

Pyroptosis, a pro-inflammatory form of regulatory cell death, is closely related to the progression of OA [44–46]. Activation of caspase-1 during pyroptosis leads to the release of inflammatory factors into the synovial fluid, intensifying the inflammatory response and ultimately causing abnormal cartilage matrix metabolism [46, 47]. It was observed that miR-203a-3p overexpression treatment significantly downregulated pyroptosis-related proteins indicating that inhibition of chondrocyte pyroptosis might be a potential target through which miR-203a-3p inhibits cartilage degeneration. In keeping with the above results, miR-203a-3p can mediate caspase 11/4-induced macrophage pyroptosis of THP-1 cells in inflammatory bowel disease [34]. Therefore, targeting the NLRP3 inflammasome could serve as a promising therapeutic strategy for OA. Our *in vitro* studies also demonstrated that miR-203a-3p mimic inhibited MDA expression and intercellular ROS generation, and increased the SOD expression, indicating that miR-203a-3p overexpression treatment alleviated LPS-induced oxidative stress in chondrocytes. Overall, oxidative stress, apoptosis, and pyroptosis of chondrocytes contribute to cartilage or synovial inflammation, exacerbate cartilage matrix degradation, and worsen OA development [40, 44]. Therefore, miR-203a-3p alleviates the pathological process of OA by regulating chondrocyte oxidative stress, apoptosis, pyroptosis, and cartilage matrix degradation.

The NF-κB pathway is a classic inflammatory pathway involved in OA development [14]. It aggravates the progression of OA by regulating chondrocyte death and abnormal cartilage matrix metabolism [15]. In this study, we also observed activation of the NF-κB pathway in LPS-stimulated chondrocytes. However, miR-203a-3p overexpression blocked this pathway by inhibiting IκBα phosphorylation and p65 nuclear translocation. Additionally, MYD88 serves as a central adaptor for downstream inflammatory signaling pathways of TLR and IL-1 receptor family members [16, 17]. As a crucial node in the inflammatory pathway, MYD88 is involved in all TLR-activated signaling pathways and promotes inflammation by activating the transcription factor NF-κB [48]. Previous studies have emphasized the critical role of the MYD88/NF-κB pathway in the pathological process of OA [48–50]. For instance, miR-382-3p has been found to inhibit chondrocyte inflammation through the TLR_4_/MYD88/NF-κB pathway in OA [48]. Similarly, miR-940 participates in OA pathogenesis via the MYD88/NF-κB pathway [51]. In our research, we observed upregulation of MYD88 expression in LPS-stimulated chondrocytes. Moreover, miR-203a-3p directly targets MYD88 to activate the NF-κB pathway. Further, inhibiting MYD88 significantly reversed the effects of miR-203a-3p inhibition on promoting cartilage matrix degradation, apoptosis, and pyroptosis in chondrocytes, confirming that miR-203a-3p negatively regulates MYD88 to exert its biological functions in chondrocytes. Hence, this study provided new insights into the important roles of miR-203a-3p and the MYD88/NF-κB pathway in preventing and treating OA.

In recent years, numerous studies have provided valuable insights into the regulatory relationship between the MYD88/NF-κB pathway and pyroptosis. It has been established that MYD88/NF-κB acts upstream of NLRP3 leading to the transcriptional activation of NLRP3 [52]. Upon activation, the MYD88/NF-κB pathway causes the dissociation of NF-κB from IκB, enabling NF-κB to translocate to the nucleus and trigger the transcriptional activation of the NLRP3 inflammasome. This process ultimately results in the release of IL-1β and IL-18, culminating in cell pyroptosis [46, 53]. Additionally, it has been demonstrated that quercetin can protect against macrophage pyroptosis by modulating the TLR_2_/MYD88/NF-κB pathway [54]. Moreover, another study showed that nicorandil could alleviate pyroptosis in rats with myocardial infarction by influencing the TLR_4_/MYD88/NF-κB/NLRP3 pathway [55]. The interplay between the NF-κB/NLRP3 pathways and their contribution to pyroptotic inflammation in OA chondrocytes has also been investigated [56]. Our present findings further confirmed the interaction between the NF-κB pathway and pyroptosis as mediators of OA progression. Thus, targeted inhibition of the MYD88/NF-κB pathway may offer a means to protect chondrocytes from pyroptosis. However, it should be noted that although our study provided important insights, the exact mechanisms underlying the interaction between NF-κB and NLRP3 in affecting OA progression remain unclear. Further investigation is warranted in future studies.

## CONCLUSIONS

In summary, our research showed that miR-203a-3p inhibited cartilage matrix degradation and alleviated oxidative stress, apoptosis, and pyroptosis *in vitro* and *in vivo* OA models by modulating the MYD88/NF-κB pathway. These findings demonstrated that miR-203a-3p had the potential to serve as a regulator for slowing down OA progression. Further clinical validation of miR-203a-3p is warranted.
